# International Caries Detection and Assessment System (ICDAS): A New Concept

**DOI:** 10.5005/jp-journals-10005-1089

**Published:** 2010-04-15

**Authors:** Neeraj Gugnani, IK Pandit, Nikhil Srivastava, Monika Gupta, Megha Sharma

**Affiliations:** 1 Professor, Department of Pedodontics, DAV Dental College, Yamunanagar, Haryana, India; 2 Principal, Department of Pedodontics, DAV Dental College, Yamunanagar, Haryana, India; 3 Associate Professor, Department of Pedodontics, DAV Dental College, Yamunanagar, Haryana, India; 4 Postgraduate Student, Department of Pedodontics, DAV Dental College, Yamunanagar, Haryana, India

**Keywords:** ICDAS, Noncavitated caries, DMFT.

## Abstract

Dental caries is a complex multifactorial disease of the calcified tissues of the teeth, caused by interaction of various factors including the host, agent, substrate and time as demonstrated by the Keyes circle. Detecting carious lesion at the earliest possible stage of its development is definitely helpful in appropriate treatment planning for the same. The lack of consistency among the contemporary criteria systems for detecting carious lesions limits the comparability of outcomes measured in epidemiological and clinical studies. Therefore, the ICDAS criteria was developed by an international team of caries researchers to integrate several new criteria systems into one standard system for caries detection and assessment. It is a clinical scoring system for use in dental education, clinical practice, research, and epidemiology, and provides a framework to support and enable personalized total caries management for improved long-term health outcomes.

## INTRODUCTION

The understanding of the caries process has continued to advance with the vast majority of evidence supporting caries as a dynamic process, which is affected by numerous modifiers tending to push the mineral equilibrium in one direction or another, i.e. towards remineralization or demineralization.^1^ With this greater understanding of the disease, there is a thrust to promote ‘preventative’ therapies that encourage the remineralization of noncavitated lesions resulting in inactive lesions and the preservation of tooth structure, function and esthetics.^2^ Central to this vision is the ability to detect caries lesions at an early stage and to quantify the degree of mineral loss, ensuring that the correct intervention is instigated.^3^ Till date, most studies conducted to measure the prevalence of caries had used DMFT(S)/ dmft(s) index thus allowing the recording of cavitated lesions only. Moreover, the use of WHO’s DMF index^4^ for caries recording will continue in future also due to its worldwide acceptance, convenience and the possibility to compare the past dental data with future findings, but there is a strong need to consider recording of noncavitated lesions as a relevant dental health indicator.^5^ For all of these reasons, there is a real need for a range of caries detection and quantification systems to augment the clinician’s diagnostic pathway.^2^ Various caries recording criteria have been used in the past based on histological picture,^6,7^ caries activity^8^ and descriptors, such as the iceberg diagrams,^9^ which described caries as a continuum from enamel, through dentin, to the pulp. But the impending need of an evidence-based system which would permit standardized caries detection and diagnosis in differing environments and situations led to the development of International caries detection and assessment system (ICDAS).

Hence, the ICDAS was developed to bring forward the current understanding of the process of initiation and progression of dental caries to the field of epidemiological and clinical research.^10^ This system allows us to record the severity and incidence of the caries in its continuum. The ICDAS I was developed in 2002 and was later modified to ICDAS II in 2005. The ICDAS I and II criteria incorporate concepts from the research conducted by Ekstrand et al (1995, 1997)^11,6^ and other caries detection systems described in the systematic review conducted by Ismail et al (2004).^12^

### The ICDAS II Visual Scoring Criteria: Discussion

The international caries detection and assessment system (ICDAS) was developed to provide clinicians, epidemiologists and researchers with an evidence-based system, which would permit standardized caries detection and diagnosis in different environments and situations.^13^ The ICDAS presents a new paradigm for the measurement of dental caries that was developed based upon the insights gained from a systematic review of the literature on clinical caries detection system^12^ and others sources.^6,14,15^ The members of the coordinating committee of ICDAS have attempted to include the largest input of the cariology community in the process of developing integrated criteria.^10^ The new emphasis on caries measurement and management may indicate that the dental community worldwide has started to recognize that we need new approaches in caries detection, assessment and management.^16^ The development of new technologies and applications has the potential to supplement clinical caries detection, but these assessments will have to be clinically meaningful by providing measurements over and above the rattle of the arrested initial and subclinical lesions.^13^

## ICDAS: THE SCORING SYSTEM

### Coronal Primary Caries Detection Criteria

The surface characteristics of a tooth structure determine the ICDAS measurements of potential histological depth of the carious lesions. The primary requirement for applying the ICDAS system is the examination of clean and dry teeth. Drying of the tooth surface is the key for detecting noncavitated lesions because water usually clogs the pores in the carious teeth and the similar refractive index of tooth and water obscures the detection of early white spot lesions. A ball-ended explorer is used to remove any remaining plaque and debris, and to check for surface contour, minor cavitation or sealants. The teeth should be cleaned with a toothbrush or a prophylaxis cup before the clinical examination. The use of a sharp explorer is not necessary because no additional accuracy is provided and it may damage the enamel surface covering the early carious lesions.^17^

**Figs 1A to G:**
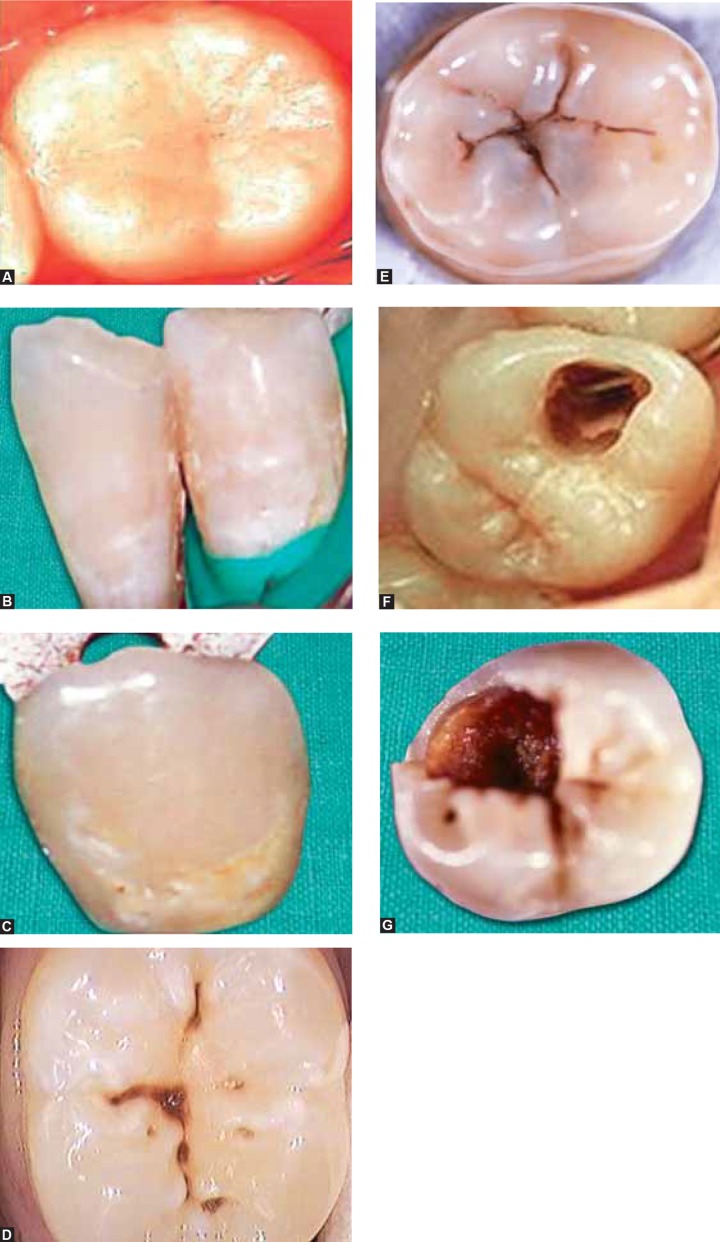
Scores of ICDAS (A) Code 0 (B) Code 1 (C) Code 2 (D) Code 3 (E) Code 4 (F) Code 5 (G) Code 6

The ICDAS detection codes for coronal caries range from 0 to 6 depending on the severity of the lesion. There are minor variations between the visual signs associated with each code depending on a number of factors, including the surface characteristics (pits and fissures versus free smooth surfaces), whether there are adjacent teeth present (mesial and distal surfaces) and whether or not the caries is associated with a restoration or sealant. Therefore, a detailed description of each of the codes is given under the following headings to assist in the training of examiners in the use of ICDAS: Pits and fissures; smooth surface (mesial or distal); free smooth surfaces and caries associated with restorations and sealants (CARS).^18^ However, the basis of the codes is essentially the same throughout (Table 1 and Figs 1A to G).

**Table 1:** **Table 1:** ICDAS II codes and criteria

*Code*		*Description*	
0		Sound tooth surface: No evidence of caries after 5 sec air drying	
1		First visual change in enamel: Opacity or discoloration (white or brown) is visible at the entrance to the pit or fissure seen after prolonged air drying	
2		Distinct visual change in enamel visible when wet, lesion must be visible when dry	
3		Localized enamel breakdown (without clinical visual signs of dentinal involvement) seen when wet and after prolonged drying	
4		Underlying dark shadow from dentine	
5		Distinct cavity with visible dentine	
6		Extensive (more than half the surface) distinct cavity with visible dentine	

## CODES DESCRIPTION

### Pit and Fissure Caries^18^

Code 0: Sound Tooth Surface

There should be no evidence of caries (either no or questionable) change in enamel translucency after prolonged air drying (suggested drying time 5 seconds). Surfaces with developmental defects, such as enamel hypoplasias, fluorosis, tooth wear (attrition, abrasion and erosion), and extrinsic or intrinsic stains will be recorded as sound. The examiner should also score as sound a surface with multiple stained fissures if such a condition is seen in other pits and fissures, a condition which is consistent with noncarious habits (e.g. frequent tea drinking).

Code 1: First Visual Change in Enamel

When seen wet there is no evidence of any change in color attributable to carious activity, but after prolonged air drying, a carious opacity or discoloration (white or brown lesion) is visible that is not consistent with the clinical appearance of sound enamel or when there is a change of color due to caries which is not consistent with the clinical appearance of sound enamel and is limited to the confines of the pit and fissure area (whether seen wet or dry). The appearance of these carious areas is not consistent with that of stained pits and fissures as defined in code 0.

Code 2: Distinct Visual Change in Enamel

The tooth must be viewed wet. When wet there is a carious opacity (white spot lesion) and/or brown carious discoloration which is wider than the natural fissure/fossa that is not consistent with the clinical appearance of sound enamel (the lesion must still be visible when dry).

Code 3: Localized Enamel Breakdown due to Caries with no Visible Dentin or Underlying Shadow

The tooth viewed wet may have a clear carious opacity (white spot lesion) and/or brown carious discoloration which is wider than the natural fissure/fossa that is not consistent with the clinical appearance of sound enamel. Once dried, there is carious loss of tooth structure at the entrance to, or within, the pit or fissure/fossa. This will be seen visually as evidence of demineralization [opaque (white), brown or dark brown walls] at the entrance to or within the fissure or pit, and although the pit or fissure may appear substantially and unnaturally wider than normal, the dentin is not visible in the walls or base of the cavity/discontinuity.

If in doubt, or to confirm the visual assessment, the WHO/CPI/PSR probe can be used gently across a tooth surface to confirm the presence of a cavity apparently confined to the enamel. This is achieved by sliding the ball end along the suspect pit or fissure and a limited discontinuity is detected if the ball drops into the surface of the enamel cavity/discontinuity.

Code 4: Underlying Dark Shadow from Dentin with or without Localized Enamel Breakdown

As a shadow of discolored dentin visible through an apparently intact enamel surface which may or may not show signs of localized breakdown (loss of continuity of the surface that is not showing the dentin). The shadow appearance is often seen more easily when the tooth is wet. The darkened area is an intrinsic shadow which may appear as grey, blue or brown in color. The shadow must clearly represent caries that started on the tooth surface being evaluated. If in the opinion of the examiner, the carious lesion started on an adjacent surface and there is no evidence of any caries on the surface being scored then the surface should be coded “0”.

Code 3 and 4, histologically may vary in depth with one being deeper than the other and vice versa. This will depend on the population and properties of the enamel. For example more translucent and thinner enamel in primary teeth may allow the undermining discoloration of the dentin to be seen before localized breakdown of enamel. However, in most cases code 4 is likely to be deeper into dentin than code 3.

Code 5: Distinct Cavity with Visible Dentin

Cavitation in opaque or discolored enamel are exposing the dentin beneath. The tooth viewed wet may have darkening of the dentin visible through the enamel. Once dried, there is visual evidence of loss of tooth structure at the entrance to or within the pit or fissure―frank cavitation. There is visual evidence of demineralization at the entrance to or within the pit or fissure and in the examiner judgment dentin is exposed.

The WHO/CPI/PSR probe can be used to confirm the presence of a cavity apparently in dentin. This is achieved by sliding the ball end along the suspect pit or fissure and a dentin cavity is detected if the ball enters the opening of the cavity and in the opinion of the examiner the base is in dentin (in pits or fissures the thickness of the enamel is between 0.5 and 1.0 mm. The deep pulpal dentin should not be probed).

Code 6: Extensive Distinct Cavity with Visible Dentin

Obvious loss of tooth structure, the cavity is both deep and wide and dentin is clearly visible on the walls and at the base. An extensive cavity involves at least half of a tooth surface or possibly reaching the pulp.

### Smooth Surface (Mesial and Distal)

This requires visual inspection from the occlusal, buccal and lingual directions.

Code 0: Sound Tooth Surface

There should be no evidence of caries (either no or questionable) change in enamel translucency after prolonged air drying. Surfaces with developmental defects, such as enamel hypoplasias; fluorosis; tooth wear (attrition, abrasion and erosion) and extrinsic or intrinsic stains will be recorded as sound.

Code 1: First Visual Change in Enamel

When seen wet there is no evidence of any change in color attributable to carious activity, but after prolonged air drying a carious opacity (white or brown lesion) is visible that is not consistent with the clinical appearance of sound enamel. This will be seen from the buccal or lingual surface.

Code 2: Distinct Visual Change in Enamel when Viewed Wet

There is a carious opacity or discoloration that is not consistent with the clinical appearance of sound enamel. This lesion may be seen directly when viewed from the buccal or lingual direction. In addition, when viewed from the occlu-sal direction, this opacity or discoloration may be seen as a shadow confined to enamel, seen through the marginal ridge.

Code 3: Initial Breakdown in Enamel due to Caries with no Visible Dentin

Once dried for approximately 5 seconds, there is distinct loss of enamel integrity, viewed from the buccal or lingual direction. If in doubt, or to confirm the visual assessment, the CPI probe can be used gently across the surface to confirm the loss of surface integrity.

Code 4: Underlying Dark Shadow from Dentin with or without Localized Enamel Breakdown

This lesion appears as a shadow of discolored dentin visible through an apparently intact marginal ridge, buccal or lingual walls of enamel. This appearance is often seen more easily when the tooth is wet. The darkened area is an intrinsic shadow which may appear as grey, blue or brown in color.

Code 5: Distinct Cavity with Visible Dentin

Cavitation in opaque or discolored enamel (white or brown) with exposed dentin in the examiner’s judgment, if in doubt, or to confirm the visual assessment, the CPI probe can be used to confirm the presence of a cavity apparently in dentin. This is achieved by sliding the ball end along the surface and a dentin cavity is detected if the ball enters the opening of the cavity, and in the opinion of the examiner the base is in dentin.

Code 6: Extensive Distinct Cavity with Visible Dentin

Obvious loss of tooth structure, the extensive cavity may be deep or wide and dentin is clearly visible on both the walls and at the base. The marginal ridge may or may not be present. An extensive cavity involves at least half of a tooth surface or possibly reaching the pulp.

A simple decision tree is provided for applying the 7-code for classifying coronal tooth surfaces following the ICDAS criteria (Flow Chart 1).^18^

### Caries-AssociatedwithRestorationsandSealants(CARS) Detection Criteria

Since outer carious lesions adjacent to restorations are thought to be analogous with primary caries, the broad principles applied to the criteria for primary caries are also applied to CARS where relevant. However, it should be noted that the scientific basis for doing so has not been established and the literature in the area of secondary caries is far more limited than that for primary coronal caries.^16^

Code 0: Sound Tooth Surface with Restoration or Sealant

A sound tooth surface adjacent to a restoration/sealant margin, there should be no evidence of caries. Surfaces with marginal defects less than 0.5 mm in width, developmental defects, and extrinsic or intrinsic stains will be recorded as sound. Stained margins consistent with noncarious and which do not exhibit signs consistent with demineralization should be scored as sound.

Code 1: First Visual Change in Enamel

When seen wet there is no evidence of any change in color attributable to carious activity, but after prolonged air drying an opacity or discoloration consistent with demineralization is visible that is not consistent with the clinical appearance of sound enamel.

Code 2: Distinct Visual Change in Enamel/Dentin Adjacent to a Restoration/Sealant Margin

If the restoration margin is placed on enamel, the tooth must be viewed wet. When wet, there is an opacity consistent with demineralization or discoloration that is not consistent with the clinical appearance of sound enamel and If the restoration margin is placed on dentin, discoloration that is not consistent with the clinical appearance of sound dentin or cementum is code 2.

**Flow Chart 1:**
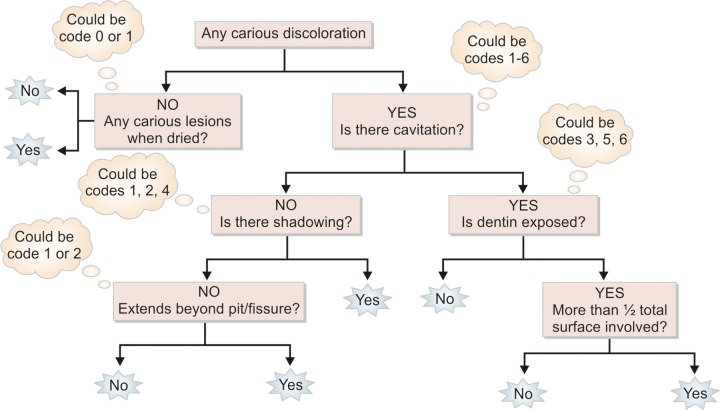
Decision tree for primary coronal caries detection

Code 3: Carious Defects of < 0.5 mm with the Signs of code 2

Cavitation at the margin of the restoration/sealant less than 0.5 mm, in addition to either an opacity or discoloration consistent with demineralization that is not consistent with the clinical appearance of sound enamel or with a shadow of discolored dentin.

Code 4: Marginal Caries in Enamel/Dentin/Cementum Adjacent to Restoration/Sealant with Underlying Dark Shadow from Dentin

The tooth surface may have characteristics of code 2 and has a shadow of discolored dentin which is visible through an apparently intact enamel surface or with localized breakdown in enamel but no visible dentin. This appearance is often seen more easily when the tooth is wet and is a darkening and intrinsic shadow which may be grey, blue, orange or brown in color.

Code 5: Distinct Cavity Adjacent to Restoration/Sealant

Distinct cavity adjacent to restoration/sealant with visible dentin in the interfacial space with signs of caries as described in code 4, in addition to a gap > 0.5 mm in width or in those instances where margins are not visible, there is evidence of discontinuity at the margin of the restoration/ sealant and tooth substance of the dentin as detected by 0.5 mm ball-ended probe run along the restoration/sealant margin.

Code 6: Extensive Distinct Cavity with Visible Dentin

Obvious loss of tooth structure, the extensive cavity may be deep or wide and dentin is clearly visible on both the walls and at the base.

### ICDAS Two-digit Coding Method

A two-number coding system is suggested to identify restorations/sealants with the first digit, followed by the appropriate caries code, e.g. a tooth restored with amalgam, which also exhibited an extensive distinct cavity with visible dentin would be coded 4 (for an amalgam restoration) and 6 (distinct cavity), an unrestored tooth with a distinct cavity would be 06. The suggested restoration/sealant coding system is as follows:^18^

  0 = Sound, i.e. surface not restored or sealed (use with the codes for primary caries)
  1 = Sealant, partial
  2 = Sealant, full
  3 = Tooth colored restoration
  4 = Amalgam restoration
  5 = Stainless steel crown
  6 = Porcelain or gold or PFM crown or veneer
  7 = Lost or broken restoration
  8 = Temporary restoration
  9 = Used for the following conditions
96 = Tooth surface cannot be examined; Surface excluded
97 = Tooth missing because of caries (tooth surfaces will be coded 97)
98 = Tooth missing for reasons other than caries (all tooth surfaces will be coded 98)
99 = Unerupted (tooth surfaces coded 99)

### Root Caries

According to the National Institutes of Health (NIH), consensus development conference on dental caries diagnosis and management, it was concluded that there was insufficient evidence on the validity of clinical diagnostic systems for root caries.^19^ Root caries are frequently observed near the cementoenamel junction (CEJ), although lesions can appear anywhere on the root surface. The color of the root lesions has been used as an indication of lesion activity. Active lesions have been described as being yellowish or light brown in color whereas arrested lesions appear darkly stained. However, color subsequently has been shown not to be a reliable indicator of caries activity.^20^

Codes for the Detection and Classification of Carious Lesions on the Root Surfaces^18^

One score will be assigned per root surface. The facial, mesial, distal and lingual root surfaces of each tooth should be classified as follows:

**Code E:** If the root surface cannot be visualized directly as a result of gingival recession or by gentle air-drying, then it is excluded. Surfaces covered entirely by calculus can be excluded or the calculus can be removed prior to determining the surface.

**Code 0:** The root surface neither exhibits any unusual discoloration that distinguishes it from the surrounding or adjacent root areas nor does it exhibit a surface defect either at the CEJ or wholly on the root surface. The root surface may have a natural anatomical contour or the root surface may exhibit a definite loss of surface continuity or an anatomical contour not consistent with the caries process.

**Code 1:** There is a clearly demarcated area on the root surface or at the CEJ that is discolored but there is no cavitation present (loss of anatomical contour < 0.5 mm).

**Code 2:** There is a clearly demarcated area on the root surface or at the CEJ that is discolored (light/dark brown, black) and there is cavitation (loss of anatomical contour ≥; 0.5 mm) present.

### ICDAS in Literature

Till yester years, the epidemiological surveys have mainly focused on DMFT/DMFS to evaluate the prevalence of caries. But such studies rely on recording of cavitated lesions only. While ICDAS allows the recording of both cavitated and noncavitated lesions in continuum. various studies have evaluated the feasibility of using ICDAS II in epidemiological surveys by comparing it with the WHO criteria. In an *in vivo* study conducted by Braga MM et al (2009)^21^ in Brazil, 252 children were examined by two different examiners using ICDAS II or WHO criteria. Caries prevalence and examination time was calculated using both systems and it was observed that examination by ICDAS II took twice as long as by WHO criteria. It was concluded that ICDAS II, besides providing information on noncavitated caries lesions, can also generate data comparable to previous surveys which used the WHO criteria.

Studies have also shown good inter- and intraexaminer reproducibility and the accuracy of ICDAS II in detecting occlusal caries, especially in the outer half of the enamel. An *in vitro* study was done by Diniz MB et al (2009)^22^, using 163 molars that were assessed twice by two examiners using the ICDAS II scoring and were validated histologically using Ekstrand and Lussi histological scores. The inter- and intraexaminer kappa values were 0.51 and 0.58 respectively and it was concluded that ICDAS II presented good repro-ducibility and accuracy in detecting occlusal caries. Another similar study was performed by Momeni AJ et al (2010)^23^ to evaluate intra- and interexaminer reproducibility of ICDAS II on occlusal caries diagnosis when different time intervals were allowed to elapse between examinations. Weighted kappa values for intra- and interexaminer reproducibility were 0.76 to 0.93 and it was observed that the time span did not have a major impact on assessing intra- and interexam-iner reproducibility. Shoaib L et al (2009)^24^ conducted an *in vitro* study to assess the validity and reproducibility of the ICDAS II criteria in primary teeth. The most advanced caries on the occlusal and approximal surfaces was recorded on 112 extracted primary molars followed by their sectioning and histological validation using the Downer and Ekstrand-Ricketts-Kidd (ERK) scoring systems and it was concluded that validity and reproducibility of the ICDAS II criteria were acceptable when applied to primary molar teeth.

Surveys have been done to compare the ICDAS criteria with the other conventional methods of detecting, scoring and diagnosing dental caries. Kuhnisch J et al (2008)^5^ compared the diagnostic outcome of the WHO criteria, ICDAS II criteria, laser fluorescence measurements and found good diagnostic potential of the ICDAS II criteria in comparison to the traditional WHO criteria such that ICDAS II proved helpful in detecting noncavitated caries lesions as well. The study also emphasized the limited use of DIAGNOdent in field studies, when using ICDAS method for caries recording.

Another study by Rodrigues JA et al (2008)^25^ compared the performance of fluorescence-based methods, radio-graphic examination and ICDAS II on occlusal surfaces. A total of 119 permanent molars were assessed using the laser fluorescence (LF and LFpen) and fluorescence camera (FC) devices, ICDAS II and bitewing radiographs (BW) followed by their histological validation. The sensitivities for dentine caries detection were 0.86 (FC), 0.78 (LFpen), 0.73 (ICDAS II), 0.51 (LF) and 0.34 (BW) and the specificities were 0.97 (BW), 0.89 (LF), 0.65 (ICDAS II), 0.63 (FC) and 0.56 (LFpen). Thus, it was concluded that LFpen, FC and ICDAS II presented better sensitivity and LF and BW better specificity. Also, ICDAS II combined with BW showed the best performance.

## CONCLUSIONS

International caries detection and assessment system allows us to record the severity and incidence of the caries in its continuum. It is certainly leading to a paradigm shift in the concept of recording both the cavitated and noncavitated lesions. In today’s scenario, the use of sharp explorer should certainly be discouraged for detection of dental caries as it may damage the intact enamel covering the early deminer-alization. As a consequence of this changing trend to record the noncavitated lesions in the daily practice, ICDAS would certainly promote preventative therapies worldwide that encourage the remineralization of noncavitated lesions resulting in inactive lesions and the preservation of tooth structure, function and esthetics and a much decreased DMF all-over.
